# Context-Dependent Role of NF-κB Signaling in Primary Liver Cancer—from Tumor Development to Therapeutic Implications

**DOI:** 10.3390/cancers11081053

**Published:** 2019-07-25

**Authors:** Carolin Czauderna, Darko Castven, Friederike L. Mahn, Jens U. Marquardt

**Affiliations:** Department of Medicine I, Lichtenberg Research Group for Molecular Hepatocarcinogenesis, University Medical Center of the Johannes Gutenberg University of Mainz, 55131 Mainz, Germany

**Keywords:** hepatocellular carcinoma, NF-κB signaling, hepatocarcinogenesis, chronic inflammation

## Abstract

Chronic inflammatory cell death is a major risk factor for the development of diverse cancers including liver cancer. Herein, disruption of the hepatic microenvironment as well as the immune cell composition are major determinants of malignant transformation and progression in hepatocellular carcinomas (HCC). Considerable research efforts have focused on the identification of predisposing factors that promote induction of an oncogenic field effect within the inflammatory liver microenvironment. Among the most prominent factors involved in this so-called inflammation-fibrosis-cancer axis is the NF-κB pathway. The dominant role of this pathway for malignant transformation and progression in HCC is well documented. Pathway activation is significantly linked to poor prognostic traits as well as stemness characteristics, which places modulation of NF-κB signaling in the focus of therapeutic interventions. However, it is well recognized that the mechanistic importance of the pathway for HCC is highly context and cell type dependent. While constitutive pathway activation in an inflammatory etiological background can significantly promote HCC development and progression, absence of NF-κB signaling in differentiated liver cells also significantly enhances liver cancer development. Thus, therapeutic targeting of NF-κB as well as associated family members may not only exert beneficial effects but also negatively impact viability of healthy hepatocytes and/or cholangiocytes, respectively. The review presented here aims to decipher the complexity and paradoxical functions of NF-κB signaling in primary liver and non-parenchymal cells, as well as the induced molecular alterations that drive HCC development and progression with a particular focus on (immune-) therapeutic interventions.

## 1. Introduction

Primary liver cancer (PLC), in particular hepatocellular carcinoma (HCC), are oncogenic paradigms for inflammation induced cancers [1]. As such, HCCs are characterized by a significant phenotypic and molecular heterogeneity and a particularly poor outcome. Accordingly, HCC consistently ranks among the most common and rapidly evolving cancers worldwide [2,3]. HCC develops in the milieu of a chronic inflammatory liver disease and, most commonly, liver cirrhosis with consecutive severe impairment of liver function. The major risk factors responsible for chronic liver disease, progression into cirrhosis and, ultimately, HCC are known and well characterized (e.g., chronic infections with hepatitis B (HBV) and C viruses (HCV), as well as excessive alcohol consumption). Due to increasing prevalence of obesity, metabolic liver diseases resembling non-alcoholic fatty liver disease and/or steatohepatitis (NAFLD/NASH) evolved to dominant etiological risk factors for HCC in several Western countries and show a sharp increase in HCC prevalence as well as a high number of HCCs without underlying cirrhosis [4,5]. 

Given its inflammatory background, it is well recognized that the life-time risk of cancer development in the liver is not only randomly induced by bad luck, i.e., stochastic events, but also highly dependent on (micro-)environmental factors such as exposure to the above described external stimuli [6]. Thus, preventive strategies and restoration of the dysbalanced hepatic microenvironment in the chronically diseased liver requires particular attention and may be therapeutically of most significant importance [7]. However, these observations also imply that molecular mechanisms of the primary liver disease(s) as well as the type of liver damage that shapes the chronically altered and inflamed liver microenvironment has to be appreciated to improve patient outcome [7,8]. Thus, key factors that contribute to the disruption of the liver microenvironment and create an adverse oncogenic field effect and prone malignant transformation should be in the center of research efforts [9]. Among the most prominent and well-characterized signaling pathways involved in the above described inflammation-fibrosis-cancer axis is the NF-κB pathway [10,11]. The dominant role of this pathway in liver cancer development and progression has been repeatedly demonstrated [12,13,14]. Further, (epi-)genetic alterations of NF-κB family members and target genes with subsequent aberrant transcriptional activity are among the most noticeable changes in liver cancer [1]. Furthermore, a significant interaction with all major oncogenic signaling pathways in the liver has been demonstrated and highlights the importance of this pathway for liver cancer development and progression [13]. Further, it is well recognized that NF-κB activation is involved in acquisition of pre-neoplastic (epi-)genetic alterations in the hepatic microenvironment during all stages of hepatocarcinogenesis. Herein, the pathway is critically involved in fueling cancer cells with pro-tumorigenic cytokines and oncogenic growth factors that enhance cell proliferation, survival and induces invasive properties, thus, greatly contributes to malignant transformation [14]. Additionally, in the majority of HCCs it is recognized that NF-κB signaling is closely linked to cross-talk between precancerous/cancer cells and stromal/immune cells [7]. As a consequence, activation of pro-inflammatory cytokines, as well as gain of autocrine IL6 signaling in hepatic progenitor cells, activate a wide range of effects on a resident and non-resident (e.g., immune cells) and could be considered as one of the key oncogenic drivers in HCC [15].

Most noticeably, and reflective of the phenotypic heterogeneity of HCC, activation of NF-κB is highly context-dependent and can be affected by different (micro-)environmental clues, including the type of underlying liver injury and respective target cells, thus, determining cellular composition of step-wise malignant transformation in the liver. Apart from hepatocytes and cholangiocytes, several other resident and non-resident cell types including adult stem/progenitor cells, hepatic stellate cells, as well as immune cells, may react differently on NF-κB activation and contribute to cancer development. The conflicting results are best described by elegant results from genetically modified mouse models. While results clearly demonstrate that context-dependent activation of NF-κB promotes HCC development, absence of NF-κB signaling also significantly enhanced liver cancer development [10,15]. Thus, in parallel with the beneficial effects of NF-κB, it could also negatively influence hepatocyte viability, particularly during pronounced NF-κB inhibition [14]. Several other immune-related and pro-oncogenic molecules displayed unanticipated tumor-suppressing/promoting effects upon activation, dependent on the cell type such as parenchymal and non-parenchymal cell types, as well as based on different etiological context (e.g., inflammation, fibrosis, cirrhosis) [16,17]. These studies demonstrate the complexity of molecular mechanisms influencing the development and progression of liver cancer that are exerted by epigenetic and genetic alterations and a cross-talk between microenvironment and damaged hepatocytes and/or cancer cells, respectively. Further, micro-environmental activation/repression of NF-κB, not only accelerates tumor initiation, but also promotes disease progression and seeding of distant metastasis in later disease stages [16,18]. Large scale genomic analyses demonstrated that gene-expression profiles obtained from surrounding non-tumoral liver tissue rather than signals from tumor specimens significantly predict patients’ survival [17]. Consistently, a recently identified and subsequently validated 186-gene signature was highly associated with the outcome of patients in different cohorts of HCC patients [17]. Poor-prognosis molecular profiles, as well as gene sets, were commonly associated with inflammation resembling interferon signaling, tumor necrosis factor α and, most noticeably, NF-κB, and downstream IL6 signaling [18,19].

Together, NF-κB and other immune interventions that modulate the cancer microenvironment show high promise to improve and personalize cancer therapies in liver cancer. In the here presented review, we will delineate how NF-κB signaling orchestrates and affects the inflammatory response during liver injury, chronic inflammatory cell death, development of fibrosis, and, finally, induction of hepatocarcinogenesis [14]. We will further describe potential therapeutic and/or preventive strategies that could explore modulation of NF-κB signaling. 

## 2. NF-κB Signaling in Liver Homeostasis, Inflammation and Fibrogenesis 

**Figure 1:**
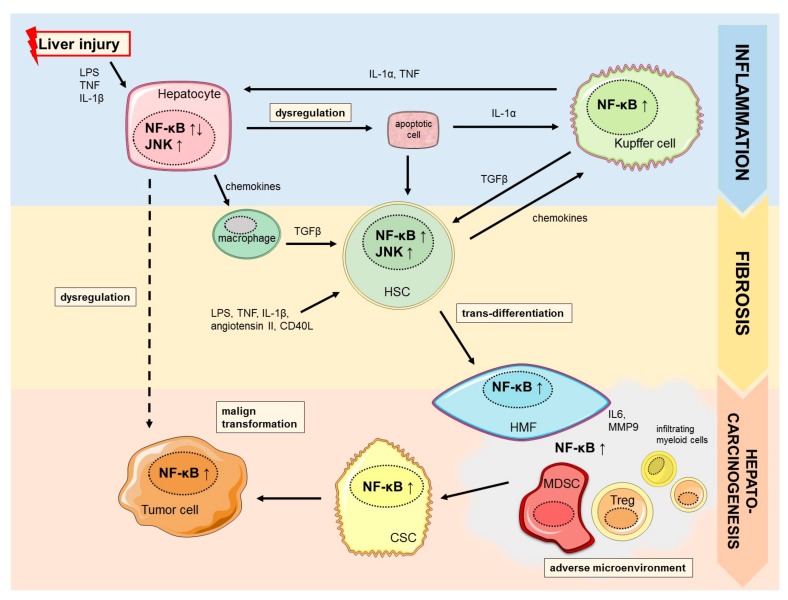
Context-dependent role of NF-κB signaling. NF-κB is involved in inflammation-fibrosis-cancer axis in a cell type dependent manner. Activated NF-κB and JNK signaling possess anti-apoptotic properties in hepatocytes upon stimulation by liver injury via LPS, TNF, and IL-1β. However, dysregulation can aggravate hepatocyte apoptosis, which subsequently stimulates Kupffer cells via IL-1α release. NF-κB activation in Kupffer cells leads to secretion of inflammatory cytokines (IL-1α, TNF) and activation of quiescent HSCs via TGFβ. NF-κB and JNK signaling further promote HSC survival and trans-differentiation to scar-forming HMFs that promote liver fibrosis in response to chronic liver damage. HMFs and invading immune cells (MDSCs, Tregs) induced by the adverse pro-oncogenic microenvironment further enhance NF-κB signaling and HCC formation. Abbreviations: CD40L, cluster of differentiation 40 ligand; CSC, cancer stem cell; HMF, hepatic myofibroblast; HSC, hepatic stellate cell; IL, interleukin; JNK, c-Jun N-terminal kinase; LPS, lipopolysaccharide; MDSC, *myeloid*-derived *suppressor cells*; *MMP9*, matrix metallopeptidase 9; NF-κB, nuclear factor κB; TGFβ, transforming growth factor β TNF, tumor necrosis factor; Treg, regulatory T cell.

Several studies provide evidence that NF-κB regulates distinct functions in diverse cell types resembling hepatocytes, Kupffer cells and hepatic stellate cells in a cell type dependent manner. Sustained or uncontrolled activation of NF-κB is linked to chronic inflammation, aggravation of liver injury and fibrosis, which is the common origin of major chronic liver diseases (Figure 1) [20]. In parenchymal cells activation of NF-κB is essential for survival whereas suppression is associated with promotion or inhibition of hepatocarcinogenesis. In contrast, NF-κB activation in Kupffer cells and hepatic stellate cells is known to promote inflammation and fibrosis during chronic inflammatory liver damage [14]. The essential function as well as controversies of NF-κB signaling in major resident and non-resident cell types during chronic liver injury and development of liver fibrosis will be discussed below. 

### 2.1. Hepato-Protective Effects of NF-κB 

A large number of factors and stimuli, such as liver injury, mediate activation of NF-κB signaling pathway. Acute liver injury can be triggered by pathogen-derived molecules (i.e., lipopolysaccharide (LPS), viral or bacterial DNA/RNA) stimulating Toll-like receptors (TLRs) or inflammatory cytokines (TNF, IL-1). Upon activation of the canonical NF-κB pathway, cytosolic NF-κB is released from its inhibitory subunits, translocates to the nucleus and induces transcription of NF-κB responsive genes [21,22]. NF-κB is known to rapidly modulate more than 200 target genes with NF-κB binding sites; mainly genes involved in regulation of inflammation, immune response, survival [23]. In order to comprehensively characterize its function in liver homeostasis, several studies used genetically modified in vivo models revealing that IKK/NF-κB system plays a critical role in hepatocyte survival [24,25,26]. Deficiency of key transcription factors such as RelA^−/−^ or cRel^−/−^ resulted in in utero death during mid-gestation due to hepatic apoptosis [24,27]. However, several genetic models with deletion of major regulating factors of NF-κB activation, e.g., IKKβ^−/−^, NEMO^−/−^ also resulted in a severe phenotype of embryonic lethality accompanied by massive hepatocyte apoptosis [25,26,28]. In order to avoid embryonic lethality, liver parenchymal cell (LPC)-specific knockout mice of different NF-κB subunits have been generated. LPC-specific knockout of NF-κB subunits (RelA/RelB/c-Rel-LPC-KO) in mice demonstrated sensitivity to tumor necrosis factor (TNF)-induced liver damage in vivo and in vitro [29,30]. Results underline the critical role of NF-κB for protection against cell death. 

Herein, anti-apoptotic functions of NF-κB are exerted by activation of anti-apoptotic target genes such as cellular inhibitors of apoptosis (cIAP1, cIAP2), XIAP, Bcl-2 family member A1 and BclXL, cFLIP, TRAF1, TRAF2, and GADD45β [23]. Moreover, during liver inflammation, stress related pathways, namely cJun (c-Jun-(N)-terminal kinase; JNK) and p38 MAPK (mitogen-activated protein kinase) kinase signaling cascades were also shown to contribute to the anti-apoptotic NF-κB response [31,32]. Recent studies further analyzed functions of NF-κB upstream components, such as the NF-κB essential modulator (NEMO), the IKK kinase complex as well as death-domain kinase receptor-interacting protein kinase 1 (RIPK1). Various studies combining LPC-specific knockout of NEMO with different death receptors including TNFR1, Fas and TRAILR showed no protection from the liver pathology induced by NEMO single knockout [33]. These findings indicated that death receptors are not essential drivers in spontaneous hepatocyte apoptosis in this model. Further, systemic lack of TNFR-1 was protective against liver damage as well as tumorigenesis [34]. However, concomitant co-deletion of death receptors only protected LPS-induced liver failure, but not spontaneous hepatocyte apoptosis in NEMO-deficient livers. In contrast, co-deletion of NEMO and FADD as well as its downstream signaling partner caspase-8 rescued mice from hepatocyte apoptosis. Thus, hepatocyte apoptosis in NEMO LPC-KO is induced by FADD- and caspase-8 dependent apoptosis [15,33,35]. Kondylis et al. showed that only impaired RIPK1 kinase activity, but not the absence of RIPK1 protein averted hepatocyte apoptosis in NEMO LPC-KO mice. Authors performed mechanistic analyses proposing that NEMO prevents degradation of major anti-apoptotic factors, i.e., cFLIP_L_, cIAP1 and TRAF2, and formation of a RIPK1/FADD/caspase-8 apoptosis-inducing complex; thereby protects hepatocytes from cell death [36]. Taken together, while a crucial role of NF-κB in protection of liver cells from pro-inflammatory cytokines is well recognized, several studies revealed previously unrecognized, NF-κB-independent roles of upstream components of the canonical NF-κB pathway on hepatic inflammation and progression to a pro-oncogenic liver damage [14,37]. A tight regulation of all components is essential to confer protection from cell death in damaged hepatocytes, while deregulation can promote chronification of the inflammatory liver damage ultimately leading to liver fibrosis and cirrhosis.

### 2.2. NF-κB in Liver Fibrogenesis

Liver fibrosis develops as the consequence of chronic liver injury and continuous inflammatory cell death. Fibrosis can regress after cessation of injury and termination of exposure of the damaging noxious agents. However, when chronic inflammatory cell death persists it may progress to cirrhosis [38]. Besides viral hepatitis and biliary or alcoholic liver disease, non-alcoholic liver steatosis (NASH) as a chronic inflammatory metabolic disease is recognized as one of the main underlying risk factors for chronic liver damage [5]. NASH incidence increases rapidly in developed countries due to a rise in obesity and metabolic syndrome [39]. Therefore, NASH has been implicated as a main contributor to the observed rising incidence of HCC in several regions of developed countries and HCC-associated NASH has become a major focus of translational research [40,41]. It has been shown that NF-κB promotes chronic inflammation of metabolic diseases as well as NASH-induced HCC [42,43,44]. Interestingly, epidemiological data revealed less activation of NF-κB signaling in normal compared to diseased livers including NASH, although a higher bacterial colonization was detected in normal livers. Results indicate a suppressed inflammation in healthy livers and underline a highly context-depending role of NF-κB-signaling [45]. As mentioned above, NF-κB possesses hepato-protective properties by preventing hepatocyte death. However, during inflammatory responses, NF-κB can also negatively impair fibrogenesis through non-parenchymal cells during inflammation. Secretion of pro-inflammatory and chemotactic factors from these non-parenchymal cells deteriorates hepatic inflammation and subsequently worsens hepatic fibrosis [14]. Sunami et al. showed that increased NF-κB signaling results in recruitment of infiltrating macrophages, promoting chronic inflammation and subsequent liver fibrosis [46]. Further, Kupffer cells, the resident macrophages in the liver, have been shown to be strong activators of NF-κB and secret pro-inflammatory chemokines and cytokines that maintain and promote inflammatory responses [20]. Furthermore, deletion of IKKβ in Kupffer cells led to a reduced production of inflammatory cytokines and mitogens such as IL6, TNF, and HGF [47]. Consistently, inhibition of NF-κB in Kupffer cells reduced degree of liver inflammation and fibrosis [48]. Upon chronic liver injury Kupffer cells also activate hepatic stellate cells (HSC) [49]. Hepatic stellate cells are other key cell types involved in deposition of extracellular matrix (ECM) components during inflammation-fibrosis-cancer-axis in the liver [20]. HSC trans-differentiate into scar-forming hepatic myofibroblasts (HMF) [50]. Survival and function of HSC/HMF are dependent on different signaling pathways including TGF-β, c-Jun/JNK as well as IKK/NF-κB-signaling [50,51]. Interestingly, inhibition of NF-κB via sulfasalazine, which blocks the activity of IKKα and IKKβ, or via IKK blocked Ser(536) phosphorylation of p65/RelA resulted in apoptosis of HMF and regression of fibrosis in vivo and in vitro [52,53]. In confirmation of the NF-κB/JNK interaction, pretreatment of HSC with the specific JNK inhibitor SP600125 was found to prevent HSC/HMF apoptosis induced by sulfasalazine [53]. The NF-κB pathway, therefore, exerts critical functions during chronic inflammation and fibrogenesis by directly affecting non-parenchymal cells and subsequent promotion of a pro-oncogenic environment.

## 3. NF-κB in Hepatocarcinogenesis

It is well recognized that chronic inflammation caused by sustained infections, autocrine and paracrine production of pro-inflammatory cytokines, as well as activation of oncogenic signaling can trigger persistent IKK activity and lead to constitutive NF-κB activation [12]. Many important aspects of tumorigenesis have been associated with NF-κB activation such as inhibition of apoptosis, cancer initiation, tumor cell proliferation, and tumor progression [54]. The majority of the studies emphasize the pro-tumorigenic role of NF-κB, particularly as a consequence of active secretion of pro-inflammatory mediators from parenchymal and non-parenchymal liver cells [14]. However, there is considerable controversy regarding the role of NF-κB in the development of HCC, as several studies also reported induction of pro-tumorigenic features upon NF-κB inactivation [15,47,55,56]. Nonetheless, it is indisputable that NF-κB can play a direct or indirect role in hepatocarcinogenesis, and that dysregulation can considerably influence clinical outcome [14,57,58].

In the above described mouse model with conditional depletion of NEMO in hepatocytes, absence of a functionally active NF-κB pathway not only aggravated inflammatory cell death, but also caused spontaneous development of liver cancer [15]. Tumor formation in the model emerged as consequence of death receptor-mediated and oxidative stress-dependent death of NEMO-deficient hepatocytes. The altered hepatic milieu concomitantly predisposed spontaneous development of chronic hepatitis and HCC development [15]. Also, MYC-induced HCC mouse model showed that specific deletion of NEMO in hepatocytes promotes hepatocarcinogenesis [59]. Interestingly, this mouse model also developed a combined hepatocellular cholangiocarcinoma (cHCC-CC), which was characterized by more aggressive and heterogeneous phenotype [59]. In line with these findings, NF-κB inhibition in hepatocytes by deletion of IKKβ was reported to have tumor-promoting properties [56]. IKKβ/NF-κB inhibition in hepatocytes was associated with increased accumulation of reactive oxygen species (ROS) and subsequent JNK and STAT3 activation [56]. A similar effect of IKKβ deficiency was observed in diethylnitrosamine (DEN) tumor model, where loss of NF-κB activity increased susceptibility to DEN-induced hepatocarcinogenesis [47,55]. Another study further showed that impaired NF-κB activation in hepatocytes upon RIPK1/TRAF2 (receptor-interacting protein kinase 1/TNF receptor-associated factor 2) inactivation led to spontaneous apoptosis and subsequently to hepatocarcinogenesis [60]. Moreover, transgenic HBsAg (HBV envelope polypeptides) mouse model, characterized by the expression of HBsAg protein and absence of HBV-specific immune response, after hepatocyte-specific inhibition of canonical NF-κB signaling showed similar effects as in previously described models [61]. Obtained results on TAK1 (MAP3-kinase TGF-β-activated kinase 1), responsible for modulation of innate and adaptive immune responses, have shown that cancer-suppressive effects of TAK1 are mediated through NF-κB activation via TNF, which consequently prevents apoptosis of hepatocytes and cholangiocytes [62]. On contrary, deficiency of TAK1 showed opposing effects, where impaired NF-κB activity lead to parenchymal cell apoptosis and hepatitis. Further development of liver injury and the onset of early HCC were the consequence of the functional gain of NEMO and hyperactivation of JNK. Altogether, these results imply that NF-κB members can exhibit opposing effects during the course of hepatocarcinogenesis, as NEMO can operate as a tumor promoter in TAK1-deficient hepatocytes independently of NF-κB. [62].

In contrast to the studies, activation of NF-κB was repeatedly demonstrated to also exert pro-tumorigenic properties. Early studies focusing on HCC development in an inflammatory context established NF-κB activation as a crucial link between inflammation and cancer [63]. A hallmark study by Pikarsky et al. showed that in *Mdr2* knockout mouse, which spontaneously develop hepatitis and liver cancer, inhibition of NF-κB signaling by overexpression of the hepatocyte-specific inducible IκB-super-repressor (non-degradable mutant form of IκBα) leads to inhibition of tumorigenesis [63]. Another mouse model that expressed lymphotoxin (LT) α,β in hepatocytes, confirmed a tumor-promoting role of NF-κB pathway [64]. Chronic liver inflammation caused by overexpression of LTα,β was followed by increased hepatocyte proliferation and subsequent HCC formation. Results suggested that IKKβ activation in hepatocytes is a significant tumor promotor that could sustain a chronic inflammatory state fueled by LTα,β expression [64,65]. More findings have shown that during chronic inflammation, B and T cells are capable of infiltrating inflamed tissue and building lymphoid aggregates, also known as ectopic lymphoid-like structures (ELS). Formation of hepatic ELS is mediated through NF-κB activation and significantly influences tumor pathogenesis, as well as patient outcome in HCC [66]. 

Several studies provided experimental evidence for a significant role of oxidative stress in regulating NF-κB signaling and promotion of hepatocarcinogenesis. A study on *Mdr2* knockout mice characterized the regulatory network of NF-κB and systematically screened for potential targets in HCC [67]. It was unveiled that co-expression of *S100A8* and *S100A9* genes during inflammation-associated hepatocarcinogenesis promoted tumor progression via ROS induction and cell death protection. Among the diverse mechanisms of ROS induction, mitochondrial fission was shown to be regularly increased in HCC tissue, which significantly influenced cell survival by promoting autophagy and preventing apoptosis. These events were mediated through ROS-dependent AKT activation and consecutive regulation of the TP53 and NF-κB pathways [68]. In addition, increased expression of Romo1 (Reactive oxygen species modulator 1) in HCC was also shown to promote production of ROS and to enhance invasiveness of HCC cells [69]. Further, tumor cell invasion by increased Romo1 expression was tightly associated with NF-κB signaling, again iterating the importance of ROS in NF-κB regulation and induction of pro-tumorigenic properties in primary liver cancer [70]. Furthermore, alcohol consumption, as one of the main predisposing factors for the development of HCC, can also act through ROS/NF-κB axis [71]. Herein, chronic alcohol consumption can induce intracellular ROS accumulation, subsequent NF-κB activation, and consequently promote angiogenesis and metastases in HCC [71]. By examining the mechanisms of angiogenesis in HCC and a role of the upregulator of cell proliferation (URGCP), Xing et al. demonstrated that this gene could significantly promote angiogenic properties of HCC cells in vitro, particularly through vascular endothelial growth factor C (VEGFC) [72]. Since *VEGFC* is a downstream target of NF-κB, it was implied that overexpression of URGCP could be responsible for upregulation of p-IKK and p-IκBα and ultimately might result in increased pathway activation [72]. In the context of NF-κB activation and malignant transformation of hepatocytes, another factor seems to play a role as the positive association between estrogen receptors (ERs) in HCV-related HCC and activated NF-κB was noted [73].

Taken together, the role of NF-κB in hepatocarcinogenesis is complex. Differential roles with regards to cancer promotion have to be perceived from different perspectives, particularly considering the context of different mouse models and types of liver injury, as well as the role of different cellular compartments of the liver. As such, various cell types play distinct roles in hepatocarcinogenesis, and that NF-κB rather acts in a cell type specific manner.

### 3.1. NF-κB in Cancer Stemness

Growing evidence assigns an important role of the NF-κB pathway in different steps of hepatocarcinogenesis by modulating cancer stem cell (CSC) features [74,75,76]. CSCs share functional properties of normal tissue stem cells such as self-renewal and differentiation capacity, and they are supposed to be exclusively responsible for tumor initiation, progression, and acquisition of chemoresistance as well as relapse formation after therapy [77,78]. The hereby defined CSC hypothesis is emerging as an alternative model of carcinogenesis, concurrent but not exclusive to the classical clonal evolution hypothesis. This hierarchic model also provides an explanation for the observed phenotypic and molecular heterogeneities in many solid tumors including liver cancer [16,79,80].

We were recently able to provide evidence for a direct involvement of NF-κB in liver cancer stemness [80]. By administration of the natural compound curcumin, an effective IKK inhibitor, as well as employment of RNAi-based inhibition of RelA, we aimed to dissect the role of NF-κB in CSCs. We could show that sensitivity to curcumin was directly related to the extent of NF-κB inhibition and downstream signaling such as JNK, Cyclin D1, and STAT3. We showed that inhibition of NF-κB in sensitive cell lines caused a dramatic decrease in CSC properties, while resistant cell lines showed enhanced CSCs characteristics. Mechanistically, NF-κB-mediated HDAC inhibition was an essential component of the CSC-depleting activity of curcumin. Accordingly, co-administration of the class I/II HDAC inhibitor trichostatine sensitized resistant cells to curcumin, indicating potential synergistic therapeutic implications. Finally, we showed that HCC patients with poor prognosis and progenitor features were most likely to benefit from NF-κB inhibition [80]. You et al. further confirmed an implication of NF-κB in liver cancer stemness [75]. They found that newly discovered protein BC047440 is highly expressed in CSCs and promotes tumor proliferation by activation of NF-κB signaling. Specific inhibition of BC047440 by shRNA led to reduction of nuclear NF-κB, and resulted in a significant reduction of CSC-related tumorigenicity [75]. Lo Re and colleagues delineated a plausible link between the stem cell regulatory properties of macroH2A1, a variant of the histone H2A and epigenetic modifier of stem-cell function, and the development of CSCs in HCC [81]. Consistently, poorly differentiated and aggressive HCC tumors possessed significantly lower level of this histone variant. By performing detailed analyses in hepatoma cell lines, authors revealed that down-regulation of macroH2A1 can induce CSC properties, such as increased expression of stemness genes, resistance to chemotherapeutic agents, higher tumorigenicity, and induction of stem-like metabolic changes. Consistently, CSC-induction was mediated through hyperphosphorylation of RelA, which led to depletion of macroH2A1 in HCC cells [81]. Another interesting study revealed significant role for the onco-protein osteopontin (OPN) in maintenance of liver cancer stemness [74,82]. Consequently, overexpression of OPN promoted development of cells with cancer stem-like characteristics, including chemoresistance, by modulating integrin αvβ3-NF-κB-hypoxia-inducible factor-1 alpha (HIF-1α) signaling axis. Several other candidate proteins were found to be involved in stemness properties of HCC, e.g., COMMD1 and COMMD7 (The COpper Metabolism MURR1 Domain (COMMD) protein family) [76]. Accordingly, COMMD7 protein was able to positively and negatively regulate NF-κB signaling in NANOG-positive CSCs by affecting NEMO sumoylation. Results also showed that increased overexpression of both proteins could lead to inhibition of NF-κB signaling. Overall, preliminary findings indicated that COMMD7 could be a potentially useful therapeutic target to improve HCC outcome by modulating CSCs via NF-κB [76]. Taken together, emerging evidence demonstrates the importance of CSCs in the initiation and progression of HCC and delineate the significant effect of NF-κB on key stemness properties [79]. Thus, modulation of the pathway might possess potential as a target for development of more efficient treatments to specifically target population of CSCs in the liver.

### 3.2. Immune Cells and NF-κB Signaling 

It is well recognized that for malignant transformation and induction of stemness in HCC the tumor microenvironment plays a critical role [83]. Chronic inflammation activates stromal cells and induces changes in the hepatic immune cell composition resulting in an adverse and immunosuppressive milieu that promotes hepatocarcinogenesis [84]. On a molecular level activation of immunosuppressive factors such as programmed death-1/programmed death ligand-1 (PD-1/PD-L1) and cytotoxic T-lymphocyte-associated protein 4 (CTLA-4) signaling occurs. This microenvironment is further promoted by infiltration of immune cells, such as myeloid-derived suppressor cells (MDSCs) and regulatory T cells (Tregs), which foster immune-escape mechanisms of cancer cells by secretion of immunosuppressive signals and cytokines [85]. Studying the role of NF-κB reveals distinct functions in stromal and immune cells. Beside activation of Kupffer cells and HSC, discussed previously, NF-κB can activate important immune functions that either impair or enhance carcinogenesis [13]. On the one hand NF-κB activation in natural killer cells controls expression of cytotoxic mediators such as perforin and granzyme B important for their antitumorigenic activity [86,87]. Activation of the canonical NF-κB pathway in T-cells also increases the amount of tumor-specific IFNγ-producing CD8+ T-cells that account for tumor elimination [88]. Importantly, disruption of the canonical NF-κB pathway in dendritic cells via the immune checkpoint molecule PD1 further results in a decrease of cytokines and expression of co-stimulatory molecules [89]. On the other hand, NF-κB has been also associated with activation of immune-suppressive effector cells in the tumor microenvironment. NF-κB, especially canonical subunit p50, has been associated in macrophage polarization and blocking NF-κB in activated tumor-associated macrophages can convert them from an immunosuppressive M2 phenotype back to M1-like cytotoxic cells [90,91]. Moreover, MDSCs can be induced through an IL-1β-induced NF-κB pathway [92]. Oh et al. recently showed that Treg development as well as their suppressor function are promoted by RelA and c-Rel [93]. However, only specific depletion of c-Rel diminished immunosuppressive activity of Treg cells and showed synergistic effects in combination with anti-PD-1 therapy [94]. Investigating the role of NF-κB signaling in the tumor microenvironment reveals distinct, complex, and highly cell type-dependent functions in immune regulation. The oncogenic impact is most likely determined by the type of liver injury and the cellular target in the tumor microenvironment. 

## 4. NF-κB as a Target for Cancer Prevention and Therapy 

Given the significant impact of NF-κB in HCC development and progression, several compounds and inhibitors have been evaluated and their underlying mechanisms have been investigated in pre-clinical settings. Studies on molecular mechanisms of approved systemic treatments with tyrosine-kinase-inhibitors (TKI), sorafenib and regorafenib, for patients with advanced HCC disease have been shown to act in part through an NF-κB-dependent mechanism: Sorafenib inhibits expressions of matrix metalloproteinase-9 (MMP9) and vascular endothelial growth factor (VEGF) by impairing the ERK/NF-κB-pathway in HCC cells [95,96]. Also, regorafenib suppresses ERK/NF-κB-activation and thereby induces extrinsic and intrinsic apoptosis in human HCC cells [97]. Furthermore, a recent study investigated mechanisms of sorafenib-resistance in HCC cell lines and revealed upregulation of immune checkpoint molecules such as PD-L1 and the epigenetic regulator, DNMT1. Both were activated through a NF-κB /STAT3-dependent manner. Results indicate that targeting the NF-κB/PD-L1/STAT3/DNMT1 axis could help to overcome acquired resistance to sorafenib treatment [98].

Interestingly, compounds used for treatment of arterial hypertension (e.g., RAS-inhibitors) or diabetes mellitus (metformin) have also been shown to possess anti-tumorigenic properties in vitro and in vivo through inactivation of NF-κB deregulation supporting their concomitant use in clinical practice [99,100,101]. Furthermore, several natural compounds have been investigated in pre-clinical models. Deoxyelephantopin naturally occurring in Chinese medicinal herbs as well as resveratrol an active polyphenol found in red wine have been shown to suppress NF-κB in vitro resulting in impaired survival and invasion properties of HCC cell lines [102,103]. Moreover, inhibition of NF-κB with curcumin, one of the most potent curcuminoids, resulted in a decrease in spherogenicity, tumorigenicity, and significant reduction of putative CSCs in HCC. The response to curcumin was associated with the inhibition of NF-κB and downstream signaling such as MYC, JNK, Cyclin D1, and STAT3 [80]. Curcumin, as well as sulforaphane, a naturally occurring isothiocyanate found in cruciferous vegetables, has been shown to further sensitize human hepatocellular carcinoma cells to radiation through inhibition of radiation-induced NF-κB activity [104,105]. Besides natural compounds, NF-κB inhibitors have also been developed and tested in pre-clinical models. Dehydroxymethylepoxyquinomicin (DHMEQ) has been derived from the structure of an antibiotic epoxyquinomicin C. Several studies evaluated the effect of DHMEQ as a NF-κB inhibitor in human hepatoma cell lines [106,107,108,109]. DHMEQ treatment dose dependently decreased the DNA-binding capacity of NF-κB RelA subunit, impaired cell viability, and induced apoptosis. Response to DHMEQ was accompanied by expression changes of genes involved in apoptosis (Bcl-XL, BAX, XIAP) and cell cycle regulation (Cyclin D1), as well as by a decrease of IL6 production [106,107]. Lampiasi et al. further observed that DHMEQ affects tumor cells more effectively in combination with celecoxib by ROS-dependent mechanisms and activation of intrinsic and extrinsic apoptotic pathways [108,109]. In vivo data on DHMEQ is restricted to a single xenograft HCC model, in which intraperitoneal administration of DHMEQ (8 mg/kg) significantly repressed the growth of Huh-7 tumor subcutaneously transplanted into BALB/c nu/nu athymic mice [106]. Another in vivo study evaluated the effect of the NF-κB inhibitor nafamostat mesylate alone or in combination with adenoviral vector-expressing tumor necrosis factor (TNF)-α in an HCC xenograft model. Huh-7 and Hep3B cells were subcutaneously injected and mice were either treated by (i) intraperitoneal (IP) injections of nafamostat mesylate, or by (ii) intratumoral (IT) injections of the human TNF-α-expressing adenoviral vector, or (iii) by a combination of nafamostat mesylate (IP) and TNF-α-expressing adenoviral vector (IT). Combined therapy resulted in a more effective reduction of tumor volume and weight compared to monotherapies. Nafamostat mesylate inhibited TNF-α–induced NF-κB activation and enhanced TNF-α-dependent caspase-8–mediated apoptosis in vitro and in vivo [110]. However, inhibitors of NF-κB have not been successfully translated for cancer therapies in humans thus far. In addition, in vivo studies centered on NF-κB signaling underline the highly context and cell type dependent role of NF-κB [15,47]. Since NF-κB in HSC/HMF induced hepatofibrogenesis favoring a pro-oncogenic micromilieu, it could be a promising preventive and/or therapeutic approach. However, clinically relevant inhibitors of this pathway are so far unspecific and cannot be applied in a cell type-specific manner. Further, pan-inhibition could result in contrary effects by affecting NF-κB signaling in cancer-depleting cells, especially immune cells [13]. NF-κB also possesses important general immune-modulatory functions by regulating immune cells and its absence could result in severe immunodeficiency [20]. Therefore, long-term pan-inhibition of NF-κB could increase the risk of infections. Importantly, our knowledge on NF-κB in liver diseases and HCC development is mainly based on in vitro and in vivo models, which do not accurately reflect HCC evolution in human liver diseases. Therefore, translation of findings on NF-κB inhibition into humans must be performed with great caution and should be evaluated and confirmed in authentic human patients and, more importantly, in a clinical setting.

## 5. NF-κB Signaling and Cholangiocarcinoma

As previously discussed, it is evident that the NF-κB signaling pathway has important implications in hepatocarcinogenesis. However, its role in the initiation and progression of intrahepatic cholangiocarcinoma (iCCA), the second most common primary liver cancer is less well explored [1]. Following distinct molecular profiles of this malignancy, many studies tried to define different subtypes of iCCA [111,112]. In this context, integrative genomic analysis describing two distinct biological classes of iCCAs, inflammatory and proliferative, identified activation of NF-κB pathway members in a small proportion of patients (10%) [113]. Despite the fact that only a small proportion of iCCA patients are affected by dysregulation in NF-κB signaling, it is still crucial to better understand the complexity of NF-κB regulation in these patients, as it might influence future (immunotherapeutic) treatment approaches. 

Similar to HCC, chronic inflammation is one of the most important predisposing factors in the development of iCCA [114]. In a subgroup of the patients harboring a poor prognosis, it was noted that genes regulating inflammation were significantly enriched [115]. During sustained inflammatory processes, cancer cell plasticity and physiology can be affected, and consequently, a more invasive phenotype can be acquired [116]. Such dramatic changes in tumor cell physiology might be a consequence of NF-κB activation in iCCA and might be induced upon exposure to TNFα, where stimulation leads to increased matrix metalloproteinase (MMP9) expression, an essential enzyme for tumor invasion and metastases [116]. Periductal inflammation and fibrosis are frequently observed during liver fluke infection, and they are important predisposing factors for development of CCA [117]. An early study investigated the expression of NF-κB in liver fluke-associated cholangiocarcinoma and showed that NF-κB family of transcription factors (p50, p52 and RelA) were highly expressed in CCA patient tissues, while the expression in normal bile duct epithelium was absent [117]. This observation was further experimentally validated in vitro and in vivo, whereas inhibition of NF-κB effectively induced apoptosis and suppressed growth in CCA cell lines, xenograft mouse model, and patient tissue. In the context of dysregulation in the innate immune response, and the pathogenesis and invasiveness of CCA, Liu and colleagues investigated the role of TLR2 and NF-κB signaling [118]. They proposed a mechanism where proliferation and invasion of CCA was mediated through the activation of NF-κB signaling and release of pro-inflammatory cytokines. The study further showed that TLR2 mediated induction of EMT markers, activation of NF-κB and upregulation of the pro-inflammatory cytokines TNF, IL6, and IL-1β [118]. Further evidence for the significance of NF-κB in CCA emerged as O-GlcNAcylation came in focus as potential therapeutic target [119,120]. Consistently, migration and invasion of CCA cells was significantly enhanced by both O-GlcNAcylation and nuclear translocation of RelA, which further promoted transcriptional activation of MMPs [120]. The role of miRNAs in the regulation of tumorigenic properties through NF-κB signaling in CCA has also been noted [121]. Consistently, miRNAs can affect iCCAs by promoting adverse properties, reflected in EMT activation and increased expression of stemness genes [121]. However, in contrast to commonly observed positive association between miRNAs and NF-κB in HCC, some cholangiocarcinomas display opposing effects of specific miRNAs (e.g., miRNA-200c, miRNA-141) and NF-κB, in the context of (cancer)stemness and poor prognosis of the patients [121]. Together, most of the existing data confirm that activated NF-κB has pro-tumorigenic function and plays an important role in tumor progression. Altogether, NF-κB or associated downstream regulators could be viable targets in CCAs. 

### Targeting NF-κB in Cholangiocarcinoma

Similar to the results obtained in HCC, curcumin as an effective IKK inhibitor was also tested on human CCA cells in order to assess potential therapeutic implications [122]. Consistently, curcumin effectively demonstrated anti-proliferative and pro-apoptotic effects through modulation of several signaling pathways with predominant inhibition of NF-κB and STAT3 signaling. Inhibition of this pathway was followed by downregulation of anti-apoptotic proteins and upregulation of PPAR-γ (peroxisome proliferator-activated receptor gamma). In addition to this, Yin et al. dissected the role of EF24 in CCA, a synthetic analog of curcumin that inhibits NF-κB signaling pathway, in vitro and in vivo [123]. The study reported that EF24 was able to effectively inhibit tumor growth and metastases by suppressing NF-κB/XIAP axis. Overall, curcumin and curcumin-based molecules again exhibited positive effects against liver malignancies and opened a new avenue for planning effective anti-cancer treatments [122]. However, as already discussed the bioavailability of the compound in vivo is decisively low [124]. Other molecular molecules have been identified as potential targets in CCAs, such as proteasome subunit ADRM1 [125]. Overexpressed of ADRM1 is frequently observed in human CCA tissue and has a negative association with patient prognosis [125]. Consistently, inhibition of ADRM1 by shRNA-based gene silencing as well as by specific targeting with ADRM1 inhibitor (RA190) in vitro and in vivo showed effective reduction in cell proliferation by inducing cell cycle arrest and apoptosis through NF-κB inactivation [125]. Overall, these results confirm that suppression the NF-κB pathway upon RA190 treatment could induce protective effects by promoting apoptosis. Based on current knowledge on the role of NF-κB signaling in CCA, it is apparent that activation of this pathway has a critical role in different aspects of cholangiocarcinogenesis, particularly in tumor progression and metastatic dissemination. Nonetheless, current knowledge on the detailed role of this pathway in iCCA is premature and further investigations on the molecular mechanisms of NF-κB are essential.

## 6. Outlook

Modern precision medicine approaches aim to direct specific treatments for each individual patient based on the dominant genetic alterations present in the respective tumor or other specific features. In the liver, failure of several targeted approaches is documented by multiple large randomized phase 3 trials that did not meet their primary endpoint over the recent years. One of the main obstacles for these approaches is a profound inter-patient as well as intratumoral heterogeneity [126,127,128]. A hallmark of this genetic heterogeneity is the diseased hepatic microenvironment that, together with oncogenic alterations, dictates the biological trait of a tumor and, thus, should be a prime target of individualized approaches [129]. Therefore, individualized approaches should also consider the specific immunological context of the individual patient. Significant progress to unravel the key regulatory mechanisms how inflammation induces immune-oncological signaling pathways have been made. Among those, aberrant NF-κB signaling has been repeatedly shown to impair cancer development over the last decades [1]. Collectively, existing evidence indicates that activation of NF-κB potentially is a major oncogenic driver of hepatocarcinogenesis despite controversies and pronounced influences through context of activation. Unfortunately, targeted approaches and molecular prediction of treatment response towards NF-κB and other immune-based intervention in liver cancer remains decisively challenging so far. Besides imperfect pre-clinical models the shortage of representative samples from advanced HCC stages are major limiting factors in this context. Moreover, in order to completely assess the oncogenic field effect and contributing cell types present in livers of human HCC patients regional sampling and single-cell genomic approaches of different tumor parts and, presumably more important, the invasive tumor front and microenvironment are required. Furthermore, regulation of the pathway, e.g., by epigenetic mechanisms including small non-coding RNAs is incompletely understood [130]. Nevertheless, existing data strongly indicate that NF-κB is an attractive target for both preventive strategies during chronic liver damage and therapeutic interventions in later stages. Future research tailored towards cell type-specific modulation of the signaling seems particularly attractive.
